# Metabolite Monomethyl Phthalate (MMP) Induces Oxidative Damage in Rat Erythrocytes: Role of Vitamins C and E

**DOI:** 10.3390/toxics13050379

**Published:** 2025-05-07

**Authors:** Xuxin Zhang, Xu Gao, Zhenxing Chi

**Affiliations:** 1 School of Marine Science and Technology, Harbin Institute of Technology, Weihai 264209, China; 2 Key Laboratory of Pollution Exposure and Health Intervention of Zhejiang Province, Hangzhou 310015, China

**Keywords:** monomethyl phthalate, erythrocytes, hemoglobin, oxidative stress, antioxidant

## Abstract

Dimethyl phthalate (DMP) can enter the human body and be absorbed into the bloodstream to produce monomethyl phthalate (MMP). MMP in the environment can also enter the bloodstream. However, little is known about the toxicity of the phthalate metabolite MMP in most organisms. In this study, the erythrocyte toxicity of MMP and a preventive approach were investigated using Sprague–Dawley (SD) rats as the model animal under MMP concentrations of 5–250 mg/kg (sub-chronic exposure in vivo) and 1.25–100 μg/mL (acute exposure in vitro). The experimental results indicate that the interaction of MMP with erythrocytes caused oxidative damage, which decreased the number of red blood cells and the hemoglobin content and increased the content of methemoglobin and the iron release of hemoglobin in rat blood. However, the above results were not observed when MMP directly interacted with hemoglobin. The antioxidants vitamin C and vitamin E improved the above blood indicators in rats. The results of this study provide certain theoretical guidance for the evaluation of the potential risks of phthalate metabolites.

## 1. Introduction

Phthalate esters (PAEs) are often added to plastic polymers as plasticizing agents [1], which are widely used in agriculture [2], commerce [3], and industry [4]. PAEs do not bind to the surrounding plastic matrix to form covalent bonds [5], so they are readily released from carbon chains with a high molecular mass, entering the environment and spreading in water [6,7], the atmosphere [8], and soil [9]. Dimethyl phthalate (DMP), one of the PAEs with the smallest molecular weight and simplest structure, has been identified as a priority environmental pollutant to be controlled [10]. DMP in the environment can enter the human body through inhalation, oral ingestion, and dermal contact [11]. A portion of DMP that enters the body is absorbed into the bloodstream and metabolized by endogenous esterase to produce monomethyl phthalate (MMP), which causes damage to the blood [12].

MMP is an environmental hormone that exerts hormone-like effects on normal metabolic processes in the body [13,14]. It exists in water (ng/L level) [15], microenvironmental dust (μg/g level) [16], and marine inlet sediment (ng/g level) [17]. Recently, MMP has been detected in body fluids such as urine (8.3 ng/mL) [18], semen (5.8 ug/mL) [19], serum (0.2–51.9 ng/mL) [20], and umbilical cord blood (1.89–2.07 ug/L) [21].

In recent years, the effects of MMP on the environment and organisms have attracted extensive attention. Studies on MMP mainly focus on its exposure level, toxic effects, and mechanisms of action regarding growth, development, reproduction, and genetics. There are also a few studies on MMP-induced human diseases and serum protein toxicity. At the biomolecular level, You et al. [22] documented sperm DNA damage induced by MMP, which was further supported by Rocha et al. [23], who showed that PAE metabolites such as MMP in the organism caused an increase in the level of 8-hydroxydeoxyguanosine, a biomarker of oxidative stress, indicating aggravated oxidative DNA damage. In addition, Gu et al. [24] elucidated the binding interactions between MMP and bovine serum albumin (BSA), revealing conformational changes that may underlie serum protein toxicity. For nervous tissue, prenatal exposure to MMP was linked to adverse neurodevelopmental outcomes in children, with male fetuses showing heightened sensitivity, as reported by Zheng et al. [25]. For endocrine tissue, Huang et al. [26] found that long-term exposure to environmental PAEs, especially low-molecular-weight metabolites of PAEs (e.g., MMP), affected thyroid and growth hormone levels in children. In a study of women undergoing in vitro fertilization and intracytoplasmic sperm injection, Deng et al. [27] investigated the urinary concentrations of eight PAE metabolites on the day of oocyte retrieval and found that MMP levels were associated with blastocyst quality. Ecotoxicological studies have shown that MMP disrupts metabolic pathways related to amino acid and energy metabolism in Daphnia magna [28], while in amphibian models, Mathieu-Denoncourt et al. [29] observed the downregulation of gene networks associated with gastrointestinal functions in western clawed frog embryos exposed to MMP, including those involved in processes related to digestion, gastric acid secretion, and motility.

Red blood cells (RBCs) are the most abundant cells in the blood, primarily responsible for the transport of oxygen and carbon dioxide [30]. Hemoglobin within RBCs contains heme groups, each with a ferrous ion (Fe^2^⁺) at its center, which reversibly bind oxygen molecules, facilitating oxygen delivery to tissues and carbon dioxide removal. However, if RBCs undergo hemolysis or if ferrous ions are oxidized to ferric ions (Fe^3^⁺) to form methemoglobin, the oxygen-carrying capacity of hemoglobin is significantly compromised.

Some studies have shown that PAEs can spontaneously enter and accumulate in membranes due to their strong hydrophobicity [31,32,33,34]. The penetration of PAEs may disrupt the permeability of the membrane, thereby affecting its normal physiological function. Due to the high fat solubility of MMP, it is easy for MMP present in the blood to penetrate the lipid membranes of erythrocytes, which may have toxic effects, resulting in a decrease in the oxygen-carrying capacity of erythrocytes and even triggering various related diseases [35]. However, there are almost no studies on the toxicity mechanisms of MMP on blood erythrocytes. Using Sprague–Dawley (SD) rats as model animals, the toxic effects of MMP on erythrocytes were investigated through in vivo and in vitro experiments, and the possible mechanism was explored from the perspective of oxidative stress. The current study helps to further highlight the toxicity of phthalate metabolites and provides theoretical guidance for the risk assessment of PAE metabolite contamination.

## 2. Materials and Methods

### 2.1. Experimental Materials

Healthy eight-week-old male SD rats weighing approximately 200 g were purchased from a formal livestock center. MMP was purchased from Shanghai Haohong Scientific Co., Ltd., Shanghai, China. Heparin sodium, vitamin C (VC), and vitamin E (VE) were purchased from Shanghai Macklin Biochemical Technology Co., Ltd., Shanghai, China. The test kits for hemoglobin (C021-1-1), methemoglobin (A102-2-1), superoxide dismutase (SOD) (A001-1-2), catalase (CAT) (A007-1-1), and malondialdehyde (MDA) (A003-1-2) were purchased from Nanjing Jiancheng Bioengineering Institute, Nanjing, China. Other chemical reagents used were purchased from Sinopharm Chemical Reagent Co., Ltd., Shanghai, China. The animal experiments were conducted according to the protocols approved by the Experimental Animal Welfare Ethics Committee of the Harbin Institute of Technology (IACUC-2021028).

### 2.2. In Vivo Exposure Experiments

The healthy male SD rats were randomly divided into two groups. One group of rats was orally administered 0, 5, 50, or 250 mg/kg (milligrams per kilogram body weight) MMP solution prepared with corn oil (total volume 2 mL). The other group of rats was orally administered 2 mL of antioxidant solution prepared with water containing 400 mg/kg (milligrams per kilogram body weight) vitamin C and 200 mg/kg (milligrams per kilogram body weight) vitamin E. Subsequently, the rats were exposed to MMP as above. The rats were sub-chronically treated by gavage at the same time every day for 21 days. After the continuous exposure experiment, blood was collected from the tail of SD rats and placed in an anticoagulant centrifuge tube for subsequent testing.

### 2.3. In Vitro Toxicity Experiments

Toxicity test on erythrocytes: Blood samples were taken from the tails of healthy SD rats. The erythrocytes were isolated by centrifugation (355× *g*, 5 min) three times with a type 80-2 medical centrifuge (Jiangsu Xinkang Medical Equipment Co., Ltd., Taizhou, China). Then, the erythrocytes were diluted with saline to prepare a 25% erythrocyte suspension. One milliliter of erythrocyte suspension was added to each centrifuge tube, and then different amounts of MMP physiological saline solution were added. Finally, physiological saline was added to fix the volume to 2 mL. Exposure to MMP concentrations of 0, 2.5, 25, and 100 μg/mL was continued for 3 h. After exposure, erythrocyte suspensions were centrifuged (555× *g*, 5 min) to discard the supernatant, and 2 mL deionized water was added to lyse the erythrocytes for subsequent detection.

Toxicity test on hemolytic blood: The erythrocytes were separated by following the above method, and then deionized water was added to create the hemolysate sample. The supernatant was collected by centrifugation (1275× *g*, 20 min). A total of 2 mL of supernatant and 1 mL of phosphate buffer solution were added to each centrifuge tube. Then, different amounts of MMP were added, and finally, deionized water was added to fix the volume to 5 mL. Exposure to MMP concentrations of 0, 1.25, 12.5, and 50 μg/mL continued for 3 h.

### 2.4. Blood Indicator Tests

Erythrocyte count: The blood samples were collected from the tail of SD rats. The erythrocyte counts were measured using the HF-3800 animal hematology analyzer (Hanfang Medical Instrument Co., Ltd., Jinan, China).

Erythrocyte hemolysis assay: After MMP treatment, the supernatant of the erythrocytes was obtained by centrifugation, and the absorbance at 540 nm was measured. The hemolysis rate of erythrocytes was calculated by using the following formula [36]:

Relative hemolysis rate (%) = *A*/*A*_1_ × 100% (1)

*A*—absorbance of the supernatants of samples from different exposure groups at 540 nm.

*A*_1_—absorbance of the supernatants from the control samples without MMP treatment at 540 nm.

Hemoglobin content assay: The samples to be tested were thoroughly mixed with the reagent in the kit according to the method in the instructions and left for 5 min. Absorbance was determined at 540 nm using a UV-Vis spectrophotometer (Shimadzu UV-1700, SHIMADZU, Kyoto, Japan). The hemoglobin content was calculated based on the absorbance readings and the formula provided in the kit manual.

Methemoglobin content test: The samples were tested to determine absorbance after the addition of the reagent according to the instructions. Absorbance was measured using a UV-Vis spectrophotometer at the wavelength specified in the kit’s protocol. Methemoglobin content was calculated based on the absorbance readings and the formula provided in the kit manual.

Hemoglobin iron release assay: The hemolysate was prepared by taking the blood of SD rats after sub-chronic exposure to MMP for 21 days and collecting erythrocytes after in vitro MMP exposure for 3 h according to the steps detailed in Section 2.3. The hemolysate was centrifuged (1275× *g*, 20 min) twice, and then the supernatant was placed into ultrafiltration centrifugation tubes for centrifugation (2167× *g*, 20 min). Nitric acid (0.5 mL) and hydrogen peroxide (125 μL) were added to the collected filtrate, and the sample was placed into a water bath for digestion (95 °C, 3 h). Then, the solution was fixed to a volume of 10 mL, and the Fe content was determined by using inductively coupled plasma mass spectrometry (ICP-MS NexION 350D, Perkin Elmer, Waltham, MA, USA). For the hemolysate after 3 h of MMP treatment, the Fe content was determined after digestion using the above method.

### 2.5. Detection of Oxidative Stress Indicators

After sub-chronic exposure of SD rats to MMP, anticoagulated whole blood was taken from the tail of rats and placed into centrifuge tubes, and the supernatant was obtained by centrifugation (799× *g*, 15 min). Absorbance measurements were performed using a UV-Vis spectrophotometer at the specific wavelengths indicated in the respective kit protocols. The activities of CAT and SOD and the MDA content were then calculated based on the absorbance readings and the formulas provided in the kit manuals. The measurement principles were as follows:

SOD: Xanthine and xanthine oxidase systems produce superoxide anion radicals that can oxidize hydroxylamine to nitrite, which appears purplish red under the action of chromogenic agents and produces absorbance at 550 nm. SOD can inhibit superoxide anion free radicals and reduce absorbance.

CAT: The decomposition reaction of H_2_O_2_ catalyzed by CAT can be quickly stopped by adding ammonium molybdate. Undecomposed H_2_O_2_ can react with ammonium molybdate to form a yellow complex, and its absorbance can be measured at 405 nm.

MDA: MDA in lipid peroxidation products can be condensed with thiobarbituric acid to form a red product with a maximum absorption peak at 532 nm.

Erythrocytes isolated after in vivo and in vitro exposure were lysed with deionized water to obtain the erythrocyte hemolysate. The activities of SOD and CAT and the content of MDA were detected according to the above procedures and principles.

### 2.6. Statistics and Data Analysis

The data from at least three independent experiments were statistically analyzed and expressed as the mean ± standard deviation (SD). The data were analyzed via ANOVA using IBM SPSS Statistics 25 software. The statistical term * *p* < 0.05 was regarded as statistically significant, and the term ** *p* < 0.01 was considered highly significant. Two-way ANOVA was performed to assess the effects of antioxidant status (antioxidant vs. non-antioxidant) and MMP concentration (0, 5, 50, 250 mg/kg) on blood indices. Statistical significance was set at *p* < 0.05. Graphs were drawn using Origin 2018 software.

## 3. Results and Discussion

### 3.1. Toxic Effects of MMP on Erythrocytes Regarding Blood Indexes

**Figure 1:**
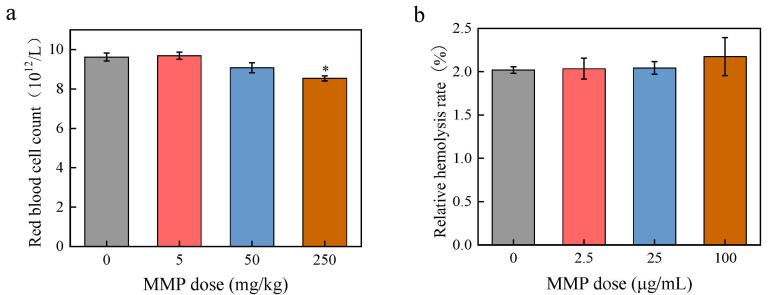
The number of erythrocytes (**a**) and the relative hemolysis rate (**b**) in the blood of rats under different MMP doses. The data are presented as the mean ± SD, *n* = 3. * *p* < 0.05 compared with the control group.

The effects of MMP on erythrocytes were observed by detecting the changes in the number of erythrocytes in the blood of rats after 21 days of in vivo MMP exposure and in the hemolysis rate of isolated erythrocytes after 3 h of in vitro MMP exposure. After 21 days of continuous MMP contamination (Figure 1a), the number of erythrocytes in the 5 mg/kg dosage group was (9.69 ± 0.18) × 10^12^/L, which was not significantly different (*p* > 0.05) from that in the control group (9.62 ± 0.20) × 10^12^/L). The number of erythrocytes in the 50 dosage group was (9.08 ± 0.25) × 10^12^/L. At an exposure dose of 250 mg/kg, the erythrocyte count in rat blood was (8.54 ± 0.13) × 10^12^/L, showing a significant decrease (* *p* < 0.05) compared to that in the control group, suggesting that MMP causes damage to erythrocytes after entering the organism.

The results of the erythrocyte hemolysis experiment are shown in Figure 1b. The rate of erythrocyte hemolysis in the control group was 2.02 ± 0.04%. The 2.5, 25, and 100 μg/mL MMP treatments resulted in an increase in the erythrocyte hemolysis rate (2.03 ± 0.12%, 2.04 ± 0.07%, and 2.17 ± 0.21%, respectively). The ANOVA results indicated no significant differences in hemolysis rates among rats exposed to different doses of MMP (*p* > 0.05).

**Figure 2:**
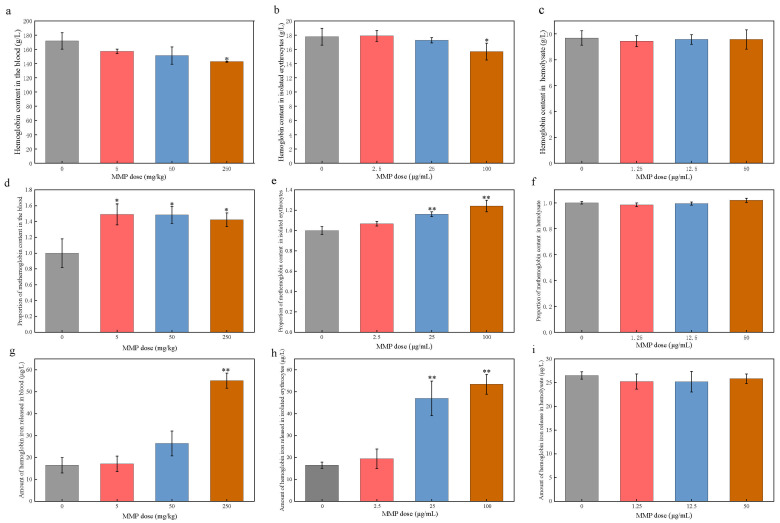
Hemoglobin content in rat blood (**a**), isolated erythrocytes (**b**), and erythrocyte hemolysate (**c**); methemoglobin content in rat blood (**d**), isolated erythrocytes (**e**), and erythrocyte hemolysate (**f**); and hemoglobin iron release in rat blood (**g**), isolated erythrocytes (**h**), and erythrocyte hemolysate (**i**) under different MMP doses. Data are presented as mean ± SD, *n* = 3. * *p* < 0.05 and ** *p* < 0.01 compared with the control group.

Using SD male rats as a model animal to investigate oral exposure to 500 mg/kg DMP for 4 weeks, Kwack et al. [37] assessed the toxicity of DMP, and their results showed that DMP caused a decrease in hemoglobin levels. However, the results were not clear for MMP, the metabolic product of DMP. Taking hemoglobin as the target, the toxic effects of MMP on erythrocytes were further investigated by detecting the changes in evaluation indexes (hemoglobin content, methemoglobin content, and hemoglobin iron release). The experimental results are shown in Figure 2.

The hemoglobin content in the blood of the rats in the 5 and 50 mg/kg dose groups was reduced (157.25 ± 3.12 g/L and 151.25 ± 12.22 g/L, respectively, vs. 171.73 ± 11.54 g/L in the control group) (Figure 2a). The hemoglobin content in the 250 mg/kg dose group (142.67 ± 0.73 g/L) showed a significant decrease (* *p* < 0.05) compared to that in the control group. This indicates that sub-chronic exposure to MMP caused a decrease in hemoglobin content in rat blood, which led to the impairment of the oxygen-carrying function of erythrocytes. The total hemoglobin content in isolated erythrocytes showed an overall decreasing trend with increasing MMP dose (Figure 2b). The 100 μg/mL MMP treatment resulted in a significant reduction in the hemoglobin content (15.69 ± 1.18 g/L vs. 17.77 ± 1.18 g/L in the control group). The hemolysate of erythrocytes contains hemoglobin. Changes in hemoglobin content after acute exposure of the erythrocyte hemolysate to MMP for 3 h (Figure 2c) were insignificant (*p* > 0.05) compared to the control group. It can be inferred that the changes in the hemoglobin content in vivo caused by MMP were due to the direct interaction of MMP with erythrocytes, not with hemoglobin.

After 21 days of sub-chronic exposure, compared with that in the control group, the content of methemoglobin in the blood of rats in the 5, 50, and 250 mg/kg MMP groups significantly increased (* *p* < 0.05) by 49.03%, 48.33%, and 42.13%, respectively (Figure 2d). After the acute exposure of erythrocytes to MMP in vitro for 3 h (Figure 2e), the content of methemoglobin in the exposure groups was higher than that in the control group, showing an increasing trend with the increase in MMP concentration. Both the 50 µg/mL and 100 µg/mL exposure groups exhibited significant increases (1.16 ± 0.02 and 1.26 ± 0.06, respectively, vs. 1.00 ± 0.04 in the control group). After 3 h of acute exposure of the erythrocyte hemolysate to MMP (Figure 2f), the methemoglobin content did not show any significant difference (*p* > 0.05) when compared to that in the control group. The direct interaction of MMP with erythrocytes, not with hemoglobin, resulted in an increase in methemoglobin content.

The release of hemoglobin iron from the blood erythrocytes of SD rats increased in a dose-dependent manner after 21 days of sub-chronic exposure to MMP (Figure 2g). A highly significant increase (** *p* < 0.01) in hemoglobin iron release was observed in the 250 mg/kg exposure group (55.09 ± 3.46 µg/L vs. 16.48 ± 3.49 µg/L in the control group). This indicates that the intake of MMP led to the release of hemoglobin iron from erythrocytes in vivo, causing damage to the oxygen-carrying function. After 3 h of acute exposure of erythrocytes to MMP in vitro (Figure 2h), the hemoglobin iron release from isolated erythrocytes was increased. The treatments with 25 μg/mL and 100 μg/mL of MMP resulted in significantly increased hemoglobin iron release (47.03 ± 7.90 µg/L and 53.43 ± 4.53 µg/L, respectively, vs. 18.51 ± 1.38 µg/L in the control group). After 3 h of exposure (Figure 2i), there was no significant change (*p* > 0.05) in the amount of hemoglobin iron released from the erythrocyte hemolysate samples under different concentrations of MMP compared with that in the control group. Erythrocytes can directly interact with MMP, leading to the release of hemoglobin iron. However, the direct interaction of MMP with hemoglobin does not lead to a reduction in iron release from hemoglobin.

In summary, it can be inferred that the direct interaction of MMP with erythrocytes after MMP enters the organism leads to erythrocyte rupture and hemolysis, causing a decrease in the number of erythrocytes. This, in turn, leads to a decrease in hemoglobin content, an increase in the content of methemoglobin, and the release of iron from hemoglobin. The direct interaction of MMP with hemoglobin is not the reason for the changes in the content of hemoglobin, methemoglobin, and iron release from hemoglobin.

### 3.2. Exploring Toxic Effects of MMP on Erythrocytes Regarding Oxidative Stress

**Figure 3:**
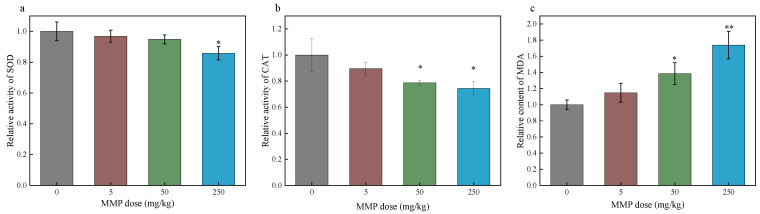
The SOD activity (**a**), CAT activity (**b**), and MDA content (**c**) in the plasma of rats after sub-chronic exposure to MMP in vivo. The data are presented as the mean ± SD, *n* = 3. * *p* < 0.05 and ** *p* < 0.01 compared with the control group.

**Figure 4:**
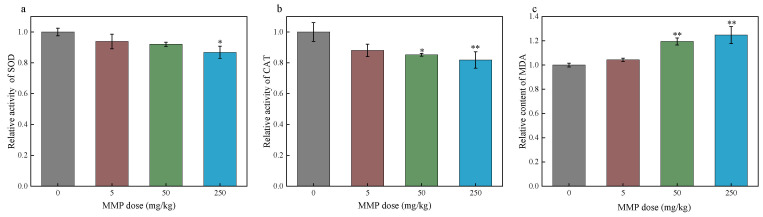
The SOD activity (**a**), CAT activity (**b**), and MDA content (**c**) of erythrocytes of rats after sub-chronic exposure to MMP in vivo. The data are presented as the mean ± SD, *n* = 3. * *p* < 0.05 and ** *p* < 0.01 compared with the control group.

**Figure 5:**
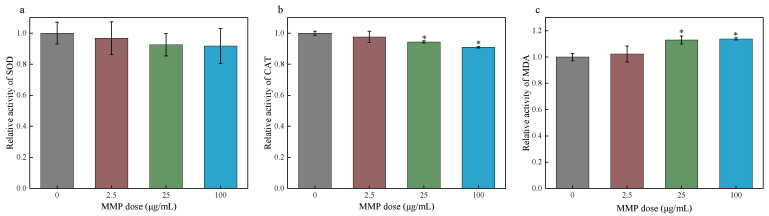
The SOD activity (**a**), CAT activity (**b**), and MDA content (**c**) in isolated erythrocytes after acute exposure to MMP in vitro. The data are presented as the mean ± SD, *n* = 3. * *p* < 0.05 compared with the control group.

Oxidative stress is defined as a transient or prolonged increase in steady-state reactive oxygen species (ROS) that cause cellular metabolic disturbances, regulatory malfunctions, and damage to cellular components [38,39]. ROS levels may increase through multiple mechanisms, including mitochondrial dysfunction, nicotinamide adenine dinucleotide phosphate (NADPH) oxidase activation, and the depletion of antioxidants [40,41,42]. When the generation of ROS surpasses the cellular antioxidant defense capacity, oxidative stress ensues [43]. Erythrocytes’ oxidative stress impairs the cell deformability necessary for efficient oxygen transport and delivery [44]. Many studies have found that PAEs and their metabolites can cause damage to the body by triggering oxidative stress [45,46,47,48]. However, further research is needed to determine whether MMP, as one of the metabolites of PAEs, can cause oxidative stress in the body. Antioxidant enzymes, including SOD and CAT, remove ROS, playing an important role in defense against oxidative stress [49]. SOD converts superoxide radicals to hydrogen peroxide, whereas CAT catalyzes the conversion of hydrogen peroxide into water [50]. GPX can catalyze the reduction of hydrogen peroxide to water and eliminate lipid peroxides (such as MDA), thereby mitigating oxidative stress [51]. ROS can attack polyunsaturated fatty acids in the cell membrane, causing lipid peroxidation and forming MDA [52,53]. When ROS generation exceeds antioxidant defenses (e.g., SOD, CAT) [54], unchecked lipid peroxidation further elevates MDA. As the final product of lipid peroxidation, MDA levels serve as a reliable indicator of the extent of oxidative damage in an organism [55]. In this study, the effects of MMP on the antioxidant capacity and oxidative stress level of blood and erythrocytes were investigated by determining the SOD and CAT activities and the MDA content of blood plasma and erythrocytes after sub-chronic MMP exposure in vivo for 21 days (Figure 3 and Figure 4) and of isolated erythrocytes after acute MMP exposure for 3 h (Figure 5).

The SOD activity in the blood plasma of rats showed a decreasing trend with the increase in MMP dose (Figure 3a). When the MMP dose was 250 mg/kg, the SOD activity in the blood plasma of rats significantly decreased by 14.20%. Compared with that in the control group, the CAT activity in the blood plasma of rats administered different MMP doses significantly decreased (Figure 3b). At MMP doses of 50 and 250 mg/kg, the CAT activity in the blood plasma of rats significantly decreased by 21.23% and 25.5%, respectively. When the MMP concentration was 50 or 250 mg/kg, the CAT activity in the blood plasma of rats significantly decreased by 21.23% or 25.5%, respectively. These results suggest that MMP can inhibit SOD and CAT activities in blood plasma and damage the antioxidant enzyme system of the organism after being ingested. The MDA content in the blood plasma of rats increased after MMP contamination (Figure 3c). At MMP doses of 50 and 250 mg/kg, MDA content significantly increased by 38.47% and 73.87%, respectively. This result indicates that MMP can lead to an increase in the level of lipid peroxidation in the blood after entering the organism, causing oxidative damage.

The SOD activity in the erythrocytes of the MMP exposure group was lower than that in the control group (Figure 4a). When the MMP dose was 250 mg/kg, the SOD activity in the blood erythrocytes of rats significantly decreased by 13.2%. The CAT activity in the blood erythrocytes of rats in the exposure group was lower than that in the control group, and when the MMP dose was 50 or 250 mg/kg, CAT activity significantly decreased by 14.8% or 18.17%, respectively (Figure 4b). This indicates that MMP can inhibit SOD and CAT activities in erythrocytes after entering the organism and damage the antioxidant enzyme system of erythrocytes. The MDA content in erythrocytes increased in the MMP-contaminated groups compared with that in the control group and tended to increase with increasing MMP dose (Figure 4c). When the MMP dose was 50 and 250 mg/kg, MDA content significantly increased by 19.43% and 24.67%, respectively. This suggests that MMP entering the organism further leads to enhanced lipid peroxidation and oxidative damage to erythrocytes.

The SOD activity in erythrocytes of the 2.5, 25, and 100 µg/mL MMP exposure groups decreased by 3.27%, 7.47%, and 8.23%, respectively, compared to that of the control group (Figure 5a). SOD activity showed a decreasing trend with the increase in MMP concentration, but the differences were not statistically significant (*p* > 0.05). The CAT activity in erythrocytes treated with MMP decreased (Figure 5b). Compared with that in the control group, at MMP exposure doses of 25 µg/mL and 100 µg/mL, the CAT activity in erythrocytes was significantly reduced by 5.67% and 9.10%, respectively. These results show that the direct effect of MMP on erythrocytes can cause a significant decrease in CAT activity and destroy the antioxidant capacity of erythrocytes. The MDA content of erythrocytes in the groups administered different MMP concentrations increased compared with that in the control group and showed a tendency to increase with the increase in MMP concentration (Figure 5c). In particular, the MDA content significantly increased by 12.97% and 13.80% at 25 and 100 µg/mL MMP concentrations, respectively, compared with that in the control group. Therefore, the direct interaction of MMP with isolated erythrocytes also caused lipid peroxidation in erythrocytes and aggravated oxidative damage to erythrocytes.

In conclusion, the decreased activities of SOD and CAT and the increased content of MDA in erythrocytes induced by MMP indicate that the direct interaction of MMP with erythrocytes after entering the body damages the antioxidant system of erythrocytes, disrupts the balance of oxidation and reduction, increases the level of lipid peroxidation, and induces oxidative stress in erythrocytes. MMP-induced oxidative stress in erythrocytes may cause a reduction in erythrocyte counts and hemoglobin levels, which is consistent with the results presented in Section 3.1.

### 3.3. Intervention with Antioxidants Against Toxic Effects of MMP on Erythrocytes

In vivo, ROS can react with nitric oxide (NO) to generate peroxynitrite, a highly reactive free radical, causing oxidative damage to cells [56]. The natural antioxidants VC and VE are important components of the non-enzymatic antioxidant system in the body [57]. VC is a potent antioxidant that plays a vital role in scavenging ROS. VC administration can successfully attenuate damage caused by oxidative stress [58]. VE is the most notable lipid-soluble antioxidant [59]. VE exerts antioxidant effects mainly through peroxygen free-radical scavenging [60]. Oxidative stress was weakened following the action of VC and VE in a rat brain cortical cellular culture [61]. In this study, the effects of MMP on erythrocytes under the intervention of antioxidants VC and VE were investigated through in vivo experiments to further explore the possible toxicity mechanisms of MMP.

**Figure 6:**
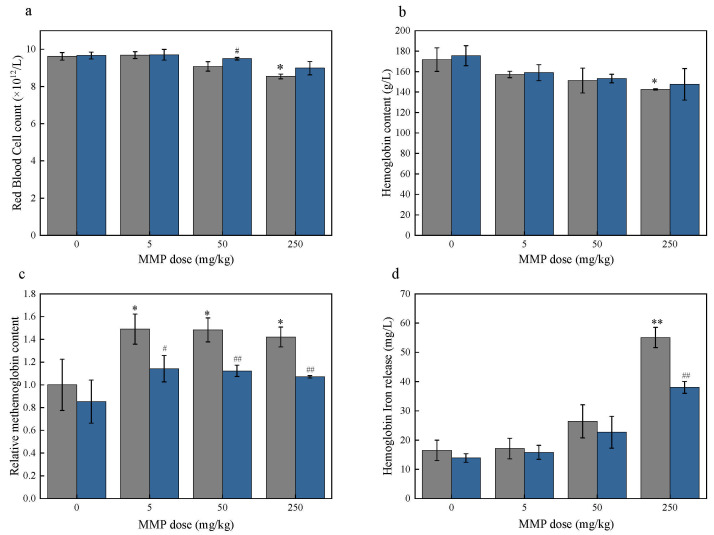
The erythrocyte count (**a**), hemoglobin content (**b**), methemoglobin content (**c**), and hemoglobin iron release amount (**d**) under different MMP doses. Blue and gray represent samples with and without antioxidant treatment, respectively. The data are presented as the mean ± SD, *n* = 3. * *p* < 0.05 and ** *p* < 0.01 compared with the control group. # *p* < 0.05 and ## *p* < 0.01 compared with the non-antioxidant group.

The number of erythrocytes in the blood of rats in the antioxidant group was higher than that in the group without the antioxidants VC and VE at the same MMP dose (Figure 6a). The number of erythrocytes in the blood of the control group in the presence of antioxidant intervention increased by 0.05 × 10^12^/L compared to that in the absence of antioxidants. Under MMP doses of 5, 50, and 250 mg/kg, the number of erythrocytes in the antioxidant groups increased by 0.01 × 10^12^/L, 0.42 × 10^12^/L, and 0.45 × 10^12^/L, respectively, compared with that in the non-antioxidant groups. The number of red blood cells in the antioxidant group with a 50 mg/kg MMP dose was significantly different (# *p* < 0.05) compared with that in the non-antioxidant group.

After MMP exposure, the hemoglobin content in the blood of rats under the intervention with antioxidants was lower than that in the control group, having the same changing tendency as the groups without antioxidant intervention (Figure 6b). Compared with the non-antioxidant groups, the hemoglobin content in the antioxidant groups increased by 1.72, 1.96, and 4.9 g/L at MMP doses of 5, 50, and 250 mg/kg, respectively.

At the same dose of MMP, the trend of changes in the methemoglobin content in the blood of rats in the antioxidant group was the same as that in the non-antioxidant group (Figure 6c). The methemoglobin content in the antioxidant group was less than that in the group without antioxidants at the same MMP dose. For MMP doses of 5, 50, and 250 mg/kg, the methemoglobin content in the antioxidant groups was reduced by 34.83%, 36.03%, and 35.07%, respectively, compared with that in the non-antioxidant groups. Compared to the non-antioxidant group, all antioxidant groups showed significant changes in methemoglobin concentration (# *p* < 0.05).

Hemoglobin iron release from the blood of rats in the antioxidant groups showed an upward trend with increasing MMP doses (Figure 6d). The amount of iron released in the presence of the antioxidant intervention was lower than that without antioxidants at the same MMP dose. The amount of iron released in the antioxidant groups was lower than that of the antioxidant-free groups by 1.31, 3.71, and 17.07 µg/L when the MMP dose was 5, 50, and 250 mg/kg, respectively. A statistically significant difference in hemoglobin iron release was observed between the antioxidant and non-antioxidant groups at an MMP dose of 250 mg/kg (## *p* < 0.01).

The above results indicate that antioxidants VC and VE were able to attenuate the effects of MMP on the number of erythrocytes, hemoglobin content, methemoglobin content, and hemoglobin iron release in the blood of rats and attenuate the toxicity of MMP toward the erythrocytes. These results further suggest that the reduction in the number of erythrocytes and the hemoglobin content, the increase in the methemoglobin content, and the release of hemoglobin iron were correlated with the oxidative stress induced by MMP.

## 4. Conclusions

Our study showed that both MMP exposure by gavage and direct interaction with erythrocytes reduced the number of erythrocytes, caused erythrocyte hemolysis, altered the hemoglobin content, and affected the content of methemoglobin and the amount of hemoglobin iron released. However, the direct effect of MMP on hemoglobin did not lead to the above content changes. This led to the conclusion that the direct interaction of MMP with erythrocytes after ingestion is one of the pathways of action that causes toxicity to erythrocytes. We investigated the effect of MMP on the antioxidant system of rat blood and erythrocytes and found that MMP inhibited the activity of antioxidant enzymes and led to oxidative stress in a dose-dependent manner. At the same MMP dose, rats with the antioxidant intervention showed improvements in all indices compared to those without antioxidant treatment. It was concluded that antioxidants could attenuate the toxic effects of MMP on erythrocytes and that there was a correlation between MMP-induced oxidative stress and the toxicity to erythrocytes it caused.

In conclusion, MMP induces oxidative stress in blood and erythrocytes, resulting in the rupture of erythrocytes, hemolysis, and a reduction in cell count, causing a decrease in hemoglobin content and an increase in the content of methemoglobin and the release of iron from hemoglobin, which may impair the oxygen-carrying, immune, and other functions of erythrocytes.

## Data Availability

The original contributions presented in this study are included in the article. Further inquiries can be directed to the corresponding author.

## Abbreviations

PAEs	Phthalate esters
DMP	Dimethyl phthalate
MMP	Monomethyl phthalate
BSA	Bovine serum albumin
RBCs	Red blood cells
SD rats	Sprague–Dawley rats
Fe^2+^	Ferrous ions
Fe^3+^	Ferric ions
VC	Vitamin C
VE	Vitamin E
SOD	Superoxide dismutase
CAT	Catalase
MDA	Malondialdehyde
SD	Standard deviation
ROS	Reactive oxygen species
NADPH	Nicotinamide adenine dinucleotide phosphate
GPX	Glutathione peroxidase
NO	Nitric oxide
